# Noninvasive Ventilation Before Intubation and Mortality in Patients Receiving Extracorporeal Membrane Oxygenation for COVID-19: An Analysis of the Extracorporeal Life Support Organization Registry

**DOI:** 10.1097/MAT.0000000000002132

**Published:** 2024-06-27

**Authors:** Marco Giani, Emanuele Rezoagli, Ryan P. Barbaro, Jordi Riera, Giacomo Bellani, Laurent Brochard, Alain Combes, Giuseppe Foti, Daniel Brodie

**Affiliations:** From the * Department of Medicine and Surgery, University of Milano-Bicocca, Monza, Italy; † Department of Emergency and Intensive Care, Fondazione IRCCS San Gerardo dei Tintori, Monza, Italy; ‡ Division of Pediatric Critical Care Medicine and Susan B. Meister Child Health Evaluation and Research (CHEAR) Center, Ann Arbor, Michigan; § Critical Care Department, Hospital Universitario Vall d’Hebron, Barcelona, Spain; ¶ SODIR, Vall d’Hebron Institut de Recerca, Barcelona, Spain; ∥ CIBERES, Instituto de Salud Carlos III, Madrid, Spain; # Department of Medical Sciences, University of Trento, Anesthesia and Intensive Care, Santa Chiara Regional Hospital, APSS Trento, Italy; ** Keenan Research Centre for Biomedical Science, Li Ka Shing Knowledge Institute, St. Michael’s Hospital, Unity Health Toronto, Toronto, Canada; †† Interdepartmental Division of Critical Care Medicine, University of Toronto, Toronto, Canada; ‡‡ Institute of Cardiometabolism and Nutrition, INSERM, Sorbonne Université, Paris, France; §§ Service de Médecine Intensive-Réanimation, Institut de Cardiologie, Hôpital Pitié-Salpêtrière, Assistance Publique-Hôpitaux de Paris, Paris, France; ¶¶ Department of Medicine, School of Medicine, Johns Hopkins University, Baltimore, Maryland.

**Keywords:** noninvasive ventilation, noninvasive respiratory support, COVID-19, acute respiratory distress syndrome, extracorporeal membrane oxygenation

## Abstract

Bilevel-positive airway pressure (BiPAP) is a noninvasive respiratory support modality which reduces effort in patients with respiratory failure. However, it may increase tidal ventilation and transpulmonary pressure, potentially aggravating lung injury. We aimed to assess if the use of BiPAP before intubation was associated with increased mortality in adult patients with coronavirus disease 2019 (COVID-19) who received venovenous extracorporeal membrane oxygenation (ECMO). We used the Extracorporeal Life Support Organization Registry to analyze adult patients with COVID-19 supported with venovenous ECMO from January 1, 2020, to December 31, 2021. Patients treated with BiPAP were compared with patients who received other modalities of respiratory support or no respiratory support. A total of 9,819 patients from 421 centers were included. A total of 3,882 of them (39.5%) were treated with BiPAP before endotracheal intubation. Patients supported with BiPAP were intubated later (4.3 *vs*. 3.3 days, *p* < 0.001) and showed higher unadjusted hospital mortality (51.7% *vs.* 44.9%, *p* < 0.001). The use of BiPAP before intubation and time from hospital admission to intubation resulted as independently associated with increased hospital mortality (odds ratio [OR], 1.32 [95% confidence interval {CI}, 1.08–1.61] and 1.03 [1–1.06] per day increase). In ECMO patients with severe acute respiratory failure due to COVID-19, the extended use of BiPAP before intubation should be regarded as a risk factor for mortality.

The landmark ARDSNet study^1^ in 2000 demonstrated that the use of low tidal volumes during invasive mechanical ventilation reduces mortality in patients with the acute respiratory distress syndrome (ARDS). Since then, high driving pressure was shown to be strongly associated with increased mortality, both during controlled^2^ and assisted mechanical ventilation.^3^ Increased attention is now focused on the potential risk of injurious ventilation and the risk of worsening lung injury during spontaneous breathing (*i.e.* patient self-inflicted lung injury [P-SILI]).^4^ To date, little attention has been paid to the potential impact of the different respiratory support (RS) modalities on outcomes, especially on mortality.

Bilevel-positive airway pressure (BiPAP, *i.e.* breathing support *via* two alternating levels of airway pressure) delivered either *via* face mask or helmet, allows to reduce respiratory effort and work of breathing. This modality of RS has proved to be beneficial in specific clinical settings, such as exacerbation of chronic obstructive pulmonary disease and ventilatory support after extubation of subsets of critically ill patients.^5,6^ However, compared to other types of noninvasive RS (*e.g.* high-flow nasal cannula [HFNC] or continuous positive airway pressure [CPAP]), BiPAP leads to an increase of tidal volumes,^7^ which may increase stress and strain on the lung and therefore worsen acute lung injury.

We hypothesized that the use of BiPAP before endotracheal intubation would be associated with increased mortality in patients with coronavirus disease 2019 (COVID-19)–related ARDS who received veno-venous extracorporeal membrane oxygenation (V-V ECMO). We gathered information from the Extracorporeal Life Support Organization (ELSO) regarding adult patients with severe respiratory failure and a COVID-19 diagnosis who underwent V-V ECMO support. The primary aim of the current study was to assess in this population if the use of BiPAP before intubation was independently associated with increased mortality assessed at hospital discharge.

## Methods

### Data Source

The ELSO Registry is an international registry with detailed information on over 185,000 ECMO cases spanning more than three decades. Within every ELSO Registry site, data managers are required to undergo a data entry exam before entering data into the registry. Detailed instructions and definitions are provided for data entry. To optimize accuracy, the registry features a point-of-entry data assessment with error and validity checks. A full record validation ensures all mandatory fields are completed at the time of record submission. Since 2020, the severe acute respiratory syndrome coronavirus 2 (SARS-CoV-2) addendum of the international registry of the ELSO includes information about preintubation RS (*i.e.*, HFNC, CPAP, BiPAP, or no RS).

The research proposal for the current study project, including a predefined statistical analysis plan, was submitted to the ELSO Scientific Oversight Committee on March 31, 2022, which approved the final version of the application on June 23, 2022. As per ELSO policy, a completely deidentified dataset was released for research purposes without the need for further ethics approval, in accordance with local regulations. The collected data included the standard information reported for all ECMO runs and additional elements from the SARS-CoV-2 addendum (see www.elso.org for data definitions).

We followed STrengthening the Reporting of OBservational studies in Epidemiology (STROBE) reporting guidelines^8^ for observational studies.

### Study Design and Patients

We included adult patients (age ≥18 years) who were diagnosed with COVID-19 *via* positive polymerase chain reaction test and were supported with ECMO with venovenous configuration for severe respiratory failure from January 1, 2020, to December 31, 2021. Patients who were not endotracheally intubated at the time of ECMO initiation were excluded. Registry follow-up data were last updated on April 15, 2022. To describe characteristics and outcomes, patients were stratified into two groups based on whether BiPAP was provided to the patient before endotracheal intubation. Patients who were not treated with BiPAP (*i.e.*, patients who received other modalities of RS—CPAP or HFNC—or no RS) were used as controls.

### Endpoints

The primary endpoint was to evaluate the independent association of BiPAP with all-cause mortality, which was assessed at hospital discharge as a dichotomous variable. Secondary outcomes were whether the time from hospital admission to intubation was associated with mortality and whether other noninvasive RS modalities may have different associations with outcome.

### Statistical Analysis

The statistical analysis was preplanned, as noted. Continuous data are reported as mean ± standard deviation or by median (interquartile range), according to the data distribution. Data normality was assessed by the Shapiro–Wilk test and by visual inspection using histograms of distribution. Categorical data are reported as count and proportion. To assess differences between characteristics and outcomes of patients who underwent BiPAP before endotracheal intubation (*i.e.*, those who were treated with BiPAP only) and controls (patients who underwent other forms of noninvasive RS, such as CPAP and HFNC, or no RS), we performed unpaired Student’s t-test or Wilcoxon–Mann-Whitney *U* test and χ^2^ test or Fisher’s exact test, for continuous and categorical variables, as appropriate.

To describe the 90 day cumulative incidence of hospital mortality over time in the study groups (*i.e.*, BiPAP *versus* controls), the Kaplan–Meier approach was used, and statistical difference between the survival curves was assessed by log-rank test.

The independent association of BiPAP with hospital mortality was evaluated with two different approaches, as follows: the first based on logistic multivariable regression models (primary analysis), and the second based on propensity score matching. Patients discharged from the ELSO center while still receiving ECMO were excluded from these analyses.

Results of multivariable logistic models were reported as odds ratios (OR) with their 95% confidence interval (95% CI). Multivariable models were adjusted for robust clustering by using the center of ECMO treatment as a cluster variable. Bilevel-positive airway pressure treatment before intubation was the exposure variable. As covariates for the multivariable analyses, we included a set of baseline characteristics (*i.e.* age, sex, race, mobile ECMO retrieval, geographic area [ELSO chapter], comorbidities, year of admission) and a set of clinically meaningful parameters gathered before the initiation of ECMO (codiagnoses [ARDS, septic shock, cardiogenic shock, pneumothorax, pneumonia, myocarditis, acute renal failure, any coinfection], treatments before ECMO [prone positioning, neuromuscular blockers, inhaled nitric oxide, steroids, renal replacement therapy, vasoactives], pH, partial pressure of arterial carbon dioxide [pCO_2_], ratio of arterial oxygen tension to the inspired fraction of oxygen [PaO_2_], positive end-expiratory pressure [PEEP], respiratory rate), ECMO referral, time from hospital admission to endotracheal intubation (which we considered as a proxy for the duration of noninvasive RS^9^) and duration of mechanical ventilation before ECMO. A further multivariable analysis was performed with the same set of covariates after excluding the—time from hospital admission to endotracheal intubation—variable (Supplemental Digital Content, http://links.lww.com/ASAIO/B192), as this information was missing for about a half of the study population.

A propensity score-matched analysis was used to compare the main outcomes between patients who received BiPAP *versus* controls. The propensity score was calculated as the predicted probability of being treated with BiPAP. The propensity score matching method was applied to estimate the effect of BiPAP *versus* other types or no RS on main outcomes. Patients were matched using the nearest neighbor approach (1:1 matching with no replacement) using a caliper of 0.2 standard deviations of the logit of the propensity score. The same baseline characteristics used for the multivariable model were included for patients matching, whereas clinical variables before the ECMO start (*e.g.*, codiagnoses at the ECMO start, time from hospital admission to endotracheal intubation, duration of mechanical ventilation before ECMO, ratio of arterial oxygen tension to inspiratory oxygen fraction (pO_2_/FiO_2_), pCO_2_, pH, respiratory rate, PEEP) were not included as they describe patient characteristics after the exposure to the study intervention (*i.e.*, after the start of noninvasive RS) and could not be considered as factors influencing the probability of being treated with BiPAP. Variable definitions are available on the ELSO website (www.elso.org). Hospital mortality between groups was compared by means of Pearson’s χ^2^ test.

As a secondary analysis, to explore the potential impact of the different RS modalities, we compared the outcomes of patients stratified into five groups, based on the type of RS used before intubation: only BiPAP, only CPAP, only HFNC, two or more modality of RS or no RS (*i.e.*, supplemental oxygen alone). Unadjusted hospital mortality was compared by means of Pearson’s χ^2^ test. Ninety day cumulative incidence of hospital mortality was described between groups by the Kaplan–Meier approach and compared by the log-rank test. The independent association of RS modality before intubation (*i.e.*, BiPAP, CPAP, HFNC, two or more RSs, no RS) and hospital mortality was then assessed by a multiple logistic regression model, to adjust for confounders at baseline, with the same set of abovementioned covariates (*i.e.*, at baseline and before the initiation of ECMO).

Statistical significance was considered with a *p* value <0.05 (two-sided). Statistical analyses and graphs were performed using STATA/MP 17.0 for MacOS (StataCorp LLC, College Station, TX).

## Results

**Figure 1.**
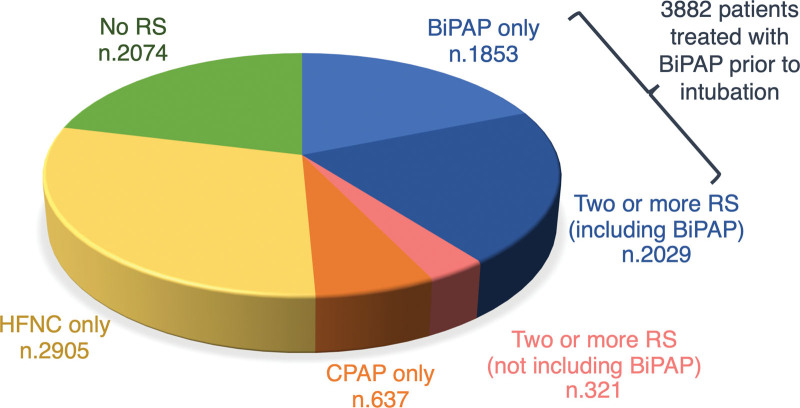
Use of the different respiratory support modalities before intubation in the study population. BiPAP, bilevel-positive airway pressure; CPAP, continuous positive airway pressure; HFNC, high-flow nasal cannula; RS, respiratory support.

From January 1, 2020, to December 31, 2021, data of 9,890 adult COVID-19 patients supported with V-V ECMO for pulmonary indications were reported to the ELSO Registry from 421 centers. Seventy-one patients were excluded because they were not endotracheally intubated at the time of ECMO cannulation. A total of 9,819 patients were then included in the current study. A total of 3,882 of them underwent BiPAP before endotracheal intubation (BiPAP group), whereas 5,937 did not (control group). Details on the RS modalities are provided in Figure 1.

Table E1 (Supplemental Digital Content, http://links.lww.com/ASAIO/B192) shows the baseline characteristics of patients and their distribution among the different ELSO Chapters.

**Figure 2.**
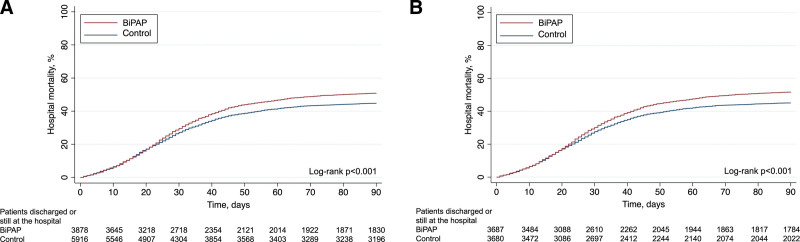
Cumulative incidence of hospital mortality at 90 day by the KM approach. Patients are stratified based on the use of noninvasive before intubation (BiPAP *vs.* controls). Mortality is displayed in the two study groups before (unadjusted KM, **A**) and after statistical adjustment (KM of propensity-matched cohorts, **B**). BiPAP, bilevel-positive airway pressure; KM, Kaplan–Meier.

Patients in the BiPAP group had a higher body mass index (BMI), differences in ethnicity and geographical distribution (*e.g.*, BiPAP support was more frequent in North America). Comorbidities were similar, except for a higher prevalence of hypertension and asthma in the BiPAP group. Patients who underwent BiPAP were intubated later than controls (4.3 [1.1–8.5] *vs.* 3.3 [0.8–7.5] days, *p* < 0.001). Overall, patients treated with BiPAP before intubation were more severe at the ECMO start, showing a higher prevalence of shock, pneumothorax, and coinfections (see Table E1, Supplemental Digital Content, http://links.lww.com/ASAIO/B192). Nitric oxide, neuromuscular blockers, steroids, and renal replacement therapy were used more frequently in patients who were treated with BiPAP before intubation, and the duration of mechanical ventilation before ECMO was higher in this group, whereas pH, pCO_2_, and pO_2_/FiO_2_ did not differ. Other information on specific coinfections, pre-ECMO treatments and parameters before and 24 hours after the start of ECMO are reported in Table E2 (Supplemental Digital Content, http://links.lww.com/ASAIO/B192). Patients in the BiPAP group showed higher unadjusted hospital mortality (52.0% *vs.* 46. 2%, *p* < 0.001, see also the cumulative incidence of mortality over time, Figure 2A), longer ECMO duration (20 *vs*. 18 days, *p* < 0.001), and higher rates of complications, such as renal failure requiring renal replacement therapy (27.3% *vs*. 24.1%, *p* < 0.001) or pneumothorax (16.7% *vs.* 12.7%, *p* < 0.001). Other complications during ECMO and outcomes are reported in Table E3 (Supplemental Digital Content, http://links.lww.com/ASAIO/B192).

A total of 2,752 patients had complete information on the abovementioned covariates. In a multivariable logistic regression analysis performed on these 2,752 patients, the use of BiPAP resulted as independently associated with higher in-hospital mortality (OR, 1.32, [95% CI, 1.08–1.61], *p* = 0.006, see Table E4, Supplemental Digital Content, http://links.lww.com/ASAIO/B192). Other factors independently associated with hospital outcome were age, sex, race, ELSO chapter, cancer, immunocompromised status, chronic heart disease, frailty, pregnancy, septic shock, cardiogenic shock and pneumothorax at the start of ECMO, acute renal failure, use of corticosteroids, hypercapnia, PaO_2_/FiO_2_, use of vasoactive agents, ECMO retrieval from another hospital, time from hospital admission to intubation (a proxy for noninvasive RS duration), and duration of mechanical ventilation before ECMO. When the same analysis was performed after excluding the—time from hospital admission to intubation—variable, a multivariable logistic regression analysis on 5,945 patients confirmed BiPAP as independently associated with hospital mortality (OR, 1.22 [95% CI, 1.05–1.42], *p* = 0.008, see Table E5, Supplemental Digital Content, http://links.lww.com/ASAIO/B192).

Propensity score matching identified 3,691 patients in each group (see Table 1 for characteristics of the matched samples). Hospital mortality of the matched samples were 53.7% and 47.0% for patients in the BIiPAP and control group, respectively (*p* < 0.001, see also Figure 2B for 90 day cumulative incidence of hospital mortality).

**Table 1.** Propensity Score Matching of Patients in the BiPAP and in the Control Group

Variable	BiPAP Group N = 3,691	Control Group N = 3,691	Standardized Difference
Propensity score	0.47 (0.38–0.53)	0.46 (0.38–0.52)	/
Baseline characteristics			
Age (years)			−0.02
Sex, female	1,137 (30.8)	1,141 (30.9)	−0.00
Comorbidities			
Hypertension	1,253 (34.0)	1,250 (33.9)	0.00
Diabetes mellitus	958 (26.0)	965 (26.1)	−0.00
Obesity	2,197 (59.5)	2,058 (55.8)	0.08
Cancer	46 (1.2)	45 (1.2)	0.00
Immunocompromised	131 (3.6)	130 (3.5)	0.00
Chronic heart disease	100 (2.7)	107 (2.9)	−0.01
Chronic renal insufficiency	97 (2.6)	96 (2.6)	0.00
Chronic lung disease	139 (3.8)	143 (3.9)	−0.00
Asthma	417 (11.3)	417 (11.3)	0.00
Frailty	16 (0.4)	22 (0.6)	−0.02
Pregnancy	141 (3.8)	150 (4.1)	−0.01
Race			
Asian	265 (7.2)	269 (7.3)	−0.00
Black	411 (11.1)	478 (13.0)	−0.06
Hispanic	781 (21.2)	820 (22.2)	−0.03
Multiple	125 (3.4)	135 (3.7)	−0.01
Other	127 (3.4)	136 (3.7)	−0.01
Unknown	143 (3.9)	147 (4.0)	−0.01
White	1,744 (47.3)	1,614 (43.7)	0.07
Middle Eastern or North African	95 (2.6)	92 (2.5)	0.01
Geographic region			
Asia-Pacific	3 (0.1)	3 (0.1)	0.00
European	481 (13.0)	495 (13.4)	−0.01
Latin-American	80 (2.2)	75 (2.0)	0.01
South and West Asia	167 (4.5)	141 (3.8)	0.04
North America	2,960 (80.2)	2,977 (80.7)	−0.01
Referral			
Not transported	901 (24.4)	915 (24.8)	−0.01
Transported on ECMO	943 (25.6)	944 (25.6)	−0.00
Transported not on ECMO	1,819 (49.3)	1,803 (48.9)	0.01
Outcome variables			
Total ECMO duration (hours)			
Overall	503 (267–859)	446 (229–809)	<0.001
Discharged alive	491 (254–909)	423 (220–829)	<0.001
Dead	514 (288–832)	478 (238–781)	0.003
ECMO outcome			
Died or poor prognosis	1,798/3,669 (49.0)	1,530/3,670 (41.7)	<0.001
Hospital length of stay (days)			
Overall	35 (22–55)	34 (21–55)	0.178
Discharged alive	46 (31–67)	43 (28–65)	0.002
Dead	27 (17–42)	26 (16–41)	0.031
Outcome at hospital discharge			<0.001
Dead	1,983 (53.7)	1,736 (47.0)	
Discharged alive	1,708 (46.3)	1,955 (53.0)	

BiPAP, bilevel-positive airway pressure; ECMO, extracorporeal membrane oxygenation.

**Figure 3.**
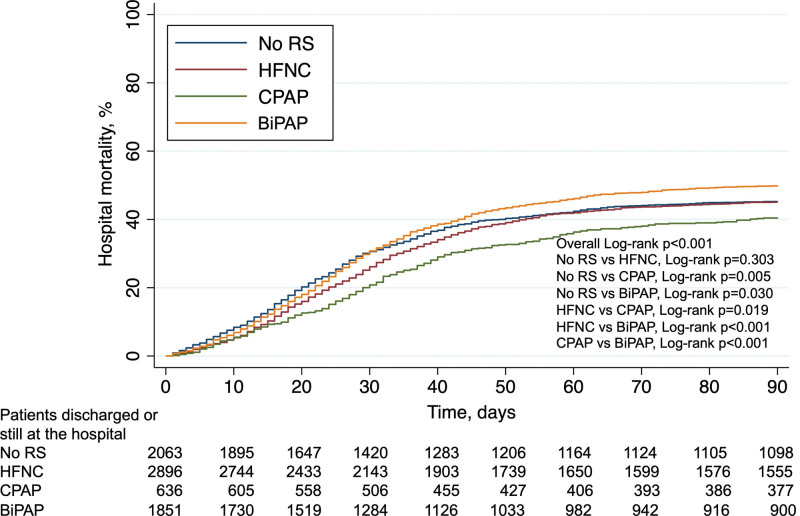
Unadjusted cumulative incidence of hospital mortality at 90 day by the KM approach. Patients are stratified based on the different noninvasive RS modalities (or no RS) before intubation. BiPAP, bilevel-positive airway pressure; CPAP, continuous positive airway pressure; HFNC, high-flow nasal cannula; KM, Kaplan–Meier; RS, respiratory support.

As a secondary analysis, we compared the outcome of the 7,469 patients who underwent only one RS modality or no RS. The unadjusted mortality of patients who underwent BiPAP was significantly higher compared to other RS modes or no RS before intubation (52.3% *vs.* 47.3 [no RS], 47.7% [HFNC], and 43.1% [CPAP], *p* value 0.002, 0.002, and <0.001, respectively, see also Figure 3).

Data from 2,043 patients were included in the multivariable logistic model for this sensitivity analysis (see Table E6, Supplemental Digital Content, http://links.lww.com/ASAIO/B192). After adjusting for baseline covariates, HFNC and no RS showed lower risk of mortality compared to BiPAP (OR, 0.66 [95% CI, 0.49–0.90] and 0.59 [0.42–0.84], respectively), whereas the difference between CPAP and BiPAP was not statistically significant (OR, 0.83 [0.56–1.22], see Table E6, Supplemental Digital Content, http://links.lww.com/ASAIO/B192).

## Discussion

In a large cohort of adult ECMO patients with COVID-19-related severe respiratory failure from the international ELSO Registry, we found an independent association between the use of BiPAP before endotracheal intubation and a higher risk of hospital mortality. To the best of our knowledge, this work represents the first large observational study investigating the potential impact of noninvasive RS modality on outcomes in patients receiving ECMO for COVID-19.

Acute respiratory distress syndrome is a clinical syndrome with numerous etiologies.^10^ Lung injury derives from three main causes: the primary infection or injury (*e.g.*, bacteria, viruses, direct trauma), the host inflammatory response, and ventilation-induced injury (*i.e.*, stress, strain). In the last 25 years, evidence has demonstrated that higher tidal volumes^1^ and higher driving pressures^2^ are associated with increased mortality in mechanically ventilated patients with ARDS. For this reason, attention is paid to limiting volumes and pressure during controlled mechanical ventilation, as tolerated. However, when the patient is spontaneously breathing (*i.e.*, during assisted mechanical ventilation), tidal volumes and driving pressures cannot be easily controlled. In this context, protective ventilation limits might not be ensured, especially in the case of low respiratory compliance.^11^ In fact, a low tidal volume is difficult to achieve in the majority of patients receiving noninvasive ventilation for *de novo* acute hypoxemic respiratory failure,^12^ and higher tidal volumes during noninvasive RS are independently associated failure of noninvasive ventilation^12,13^ and with higher 90 day mortality.^13^ Bellani *et al.*^14^ showed that, in the most hypoxic ARDS patients (*i.e.*, those with a PaO_2_ to FiO_2_ ratio below 150 mm Hg), the use of noninvasive ventilation before endotracheal intubation was associated with higher intensive care unit (ICU) mortality. Accordingly, a study by Nevola *et al.*^15^ showed that patients undergoing noninvasive support in a moderate stage (PaO_2_/FiO_2_ 101–200 mm Hg) show a significantly lower in-hospital mortality rate and length of hospitalization than those in the severe stage (PaO_2_/FiO_2_ ≤ 100 mm Hg). Wendel Garcia *et al.*^16^ and Marti et al.^17^ reported the association of noninvasive ventilation and higher overall ICU mortality in critical COVID-19 patients. Furthermore, Reyes *et al*.^18^ reported that noninvasive ventilation failure in COVID-19 patients is associated with very high mortality rates. For this reason, as highlighted by Brochard *et al*.,^4,19^ switching to controlled ventilation and neuromuscular blocker use might be indicated for severely lung-injured patients at specific points in their course to minimize progression of lung injury. To date, we lack a reliable method to predict which patients will deteriorate when noninvasive support is initiated. Consequently, we may consider limiting the duration of noninvasive support for patients with severe hypoxia (*e.g*., those with a PaO_2_ to FiO_2_ ratio below 100–150 mm Hg) who do not exhibit clinical improvement during noninvasive support. It is noteworthy that in all the analyses, the odds ratios for the time from hospital admission to intubation and for the time from intubation to ECMO (*i.e*., mechanical ventilation [MV] duration) were nearly identical (1.03/1.04 per day increase), highlighting the similar impact of spontaneous breathing duration (*i.e*., noninvasive RS duration) and invasive mechanical ventilation on outcomes.

During the SARS-CoV-2 pandemic, the use of noninvasive RS extended beyond its traditional indications,^20^ in some cases delaying endotracheal intubation and potentially increasing the risk of P-SILI. The use of noninvasive ventilation increased over time,^21,22^ and was hypothesized^23,24^ to be a contributing factor to the increasing in mortality over time noted in patients receiving ECMO for COVID-19. The findings of the current study seem to align with this hypothesis, although our data do not permit us to establish a causal relationship between BiPAP use and mortality.

Another potential determinant of P-SILI is the duration of RS before intubation (*i.e*., the “dose” of noninvasive RS). The potential impact of RS duration was recently investigated in two small cohorts of ECMO patients. Patients with a duration of RS of 3 days or more were less likely to be liberated from ECMO^25^ and experienced increased ECMO duration.^26^ Other studies showed that time from symptoms to cannulation,^27^ time from infiltrates on chest x-ray to cannulation,^28^ or time from ICU admission to endotracheal intubation^9^ (which may be a proxy for noninvasive RS duration) are independent predictors of mortality. In the present work, we included time from admission to intubation as a covariate in the multivariable analysis and found it significantly associated with increased hospital mortality.

This study has limitations. First, statistical adjustments were performed on the available characteristics, which did not include some relevant data collected before noninvasive RS, such as respiratory rate, oxygenation levels, and severity scores at hospital admission. Second, we were not able to stratify patients based on the interface used for BiPAP (*i.e*., helmet *versus* face mask), therefore we cannot exclude that different interfaces may be associated different outcomes.^29^ Third, due to data deidentification, we had no access to the admission date but only to the year of admission. For this reason, we were not able to further stratify patients on the COVID-19 pandemic waves. Fourth, using the ELSO Registry data may introduce a bias due to the missing-denominator effect, as it only includes patients who were treated with ECMO (*i.e.*, patients who failed noninvasive support and conventional mechanical ventilation). Finally, a selection bias cannot be ruled out due to the observational nature of the data. Bilevel-positive airway pressure may have been administered to patients with greater severity, and this severity may not have been entirely captured by the variables we adjusted for. However, it should be highlighted that patients in the BiPAP group were intubated later compared to controls. Furthermore, it is very difficult to envisage a randomized controlled trial on this topic; for this reason, insights from large observational studies are warranted.

## Conclusions

In a large cohort of adult patients with COVID-19–related ARDS who received V-V ECMO, the use of BiPAP was independently associated with increased in-hospital mortality. In this population, the extended use of BiPAP before intubation should be regarded as a risk factor for mortality. Although the current study has the strength of being derived from a large data set, the effect of potential residual confounders cannot be excluded.

## Supplementary Material
