# Supplementary material for: Noninvasive Ventilation Before Intubation and Mortality in Patients Receiving Extracorporeal Membrane Oxygenation for COVID-19: An Analysis of the Extracorporeal Life Support Organization Registry
Source: ASAIO J. 2024 Jun 27;70(7):633–9. doi: 10.1097/MAT.0000000000002132 (PMC11210943; doi:10.1097/MAT.0000000000002132)
Supplement: Supplementary file 1 [file mat-70-633-s001.pdf]

Online Supplement

Non-invasive ventilation prior to intubation and mortality in patients receiving ECMO for COVID-19

An analysis of the Extracorporeal Life Support Organization registry

Marco Giani †[1] M.D., Emanuele Rezoagli†[1] M.D., Ph.D., Ryan Barbaro[2] M.D., Jordi Riera[3] Ph.D., Prof. Giacomo Bellani[1] M.D., Ph.D., Prof. Laurent Brochard, M.D., Prof. Alain Combes[5] Ph.D., Prof. Giuseppe Foti*[1] M.D. and Prof. Daniel Brodie*[6] M.D.

Table E1. Description of the enrolled population stratified by utilization of Bilevel Positive Airway Pressure (BiPAP) before endotracheal intubation.

Patient characteristics (N=9819)	BiPAP group		Control group		P value
	N	Median (IQR) or n (%)	N	Median (IQR) or n (%)	
**Participants**	3882	-	5937		-
**Age, years**	3882	47 (38-55)	5937	48 (39-56)	<0.001
**Female gender**	3882	1192 (30.7)	5936	1786 (30.1)	0.515
**Weight**, kg	3866	100 (85-118)	5884	92 (80-110)	<0.001
**Height**, cm	3729	172 (165-180)	5572	170 (165-178)	<0.001
**Body-mass index**, kg/m^2	3729	33.7 (29.2-39.4)	5570	31.8 (27.7-37.0)	<0.001
**Race**	3882		5937		<0.001
Asian		283 (7.3)		558 (9.4)	
Black		428 (11.0)		637 (10.7)	
Hispanic		802 (20.7)		1288 (21.7)	
Middle Eastern or North African		98 (2.5)		297 (5.0)	
Multiple		132 (3.4)		334 (5.6)	
Other		131 (3.4)		233 (3.9)	
Unknown		145 (3.7)		238 (4.0)	
White		1863 (48.0)		2352 (39.6)	
**ELSO Chapter**	3882		5937		<0.001
Asia-Pacific		3 (0.1)		100 (1.7)	
European		485 (12.5)		1497 (25.2)	
Latin-american		80 (2.1)		493 (8.3)	
North American		3141 (80.9)		3400 (57.3)	
South and West Asia		173 (4.5)		447 (7.5)	
**Year**	3882		5937		<0.001
2020		1415 (36.5)		2982 (50.2)	
2021		2467 (63.5)		2955 (49.8)	
**Pre-ECMO comorbidities**					
Hypertension	3882	1330 (34.3)	5937	1826 (30.8)	<0.001
Diabetes	3882	996 (25.7)	5937	1435 (24.2)	0.095
Obesity	3882	2323 (59.8)	5937	2868 (48.3)	<0.001
Cancer	3882	48 (1.2)	5937	85 (1.4)	0.413
Immunocompromised	3882	135 (3.5)	5937	208 (3.5)	0.946
Pre-existing heart disease	3882	104 (2.7)	5937	168 (2.8)	0.656
Pre-existing renal insufficiency	3882	98 (2.5)	5937	150 (2.5)	0.995
Pre-existing lung disease	3882	151 (3.9)	5937	190 (3.2)	0.068
Asthma	3882	443 (11.4)	5937	593 (10.0)	0.025
Frailty	3882	29 (0.8)	5937	27 (0.5)	0.060
Pregnancy	3882	144 (3.7)	5937	263 (4.4)	0.080
**Time from hospital admission to intubation,** days	1449	4.3 (1.1-8.5)	2265	3.3 (0.8-7.5)	<0.001
**Duration of mechanical ventilation before ECMO,** days	3444	2.9 (1.0-5.5)	5064	3.5 (1.1-6.4)	<0.001
**Acute Co-diagnosis at the ECMO start**					

ARDS	3882	3297 (84.9)	5937	4762 (80.2)	<0.001
Septic Shock	3882	1007 (25.9)	5937	1360 (22.9)	0.001
Heart Failure	3882	176 (4.5)	5937	222 (3.7)	0.051
Pneumothorax	3882	733 (18.9)	5937	816 (13.7)	<0.001
Pneumonia	3882	2484 (64.0)	5937	3440 (57.9)	<0.001
Myocarditis	3882	18 (0.5)	5937	36 (0.6)	0.350
Acute Renal Failure	3882	1001 (25.8)	5937	1430 (24.1)	0.056
Any co-infection	3882	2150 (55.4)	5937	2799 (47.2)	<0.001
**Pre-ECMO treatments**					
Prone Positioning	3882	2285 (58.9)	5937	3389 (57.1)	0.081
Neuromuscular blockers	3882	2913 (75.0)	5937	4293 (72.3)	0.003
Inhaled nitric oxide	3882	621 (16.0)	5937	820 (13.8)	0.003
Steroids	3882	1933 (49.8)	5937	2406 (40.5)	<0.001
Renal replacement therapy	3882	171 (4.4)	5937	335 (5.6)	0.007
Vasoactives	3882	2120 (54.6)	5937	3366 (56.7)	0.042
**Blood gas and acid base before ECMO**					
pH	3276	7.3 (7.21-7.37)	5034	7.30 (7.21-7.37)	0.230
pCO_2, mmHg	3236	61 (51-75)	4914	61 (51-75)	0.771
pO_2/FiO_2, mmHg	3064	69 (56-87)	4700	70 (58-90)	0.316
**Ventilatory setting before ECMO**					
Respiratory rate, breaths/min	3297	28 (22-30)	4733	26 (22-30)	0.001
Positive end-expiratory pressure, cmH_2O	3280	14 (12-16)	4790	14 (10-16)	<0.001

Data are presented as frequency and percentage for qualitative variables and as median (25th-75th percentile) for quantitative variables. BiPAP, bilevel positive airway pressure ; ECMO, extracorporeal membrane oxygenation; RS: respiratory support;. HFNC, high flow nasal cannulae; CPAP, continuous positive airway pressure; RS, respiratory support; ARDS, acute respiratory distress syndrome; pCO_2, arterial carbon dioxide tension; pO_2/FiO_2, ratio of arterial oxygen tension to inspiratory oxygen fraction.

Table E2 Co-infections, pre-ECMO treatments and parameters before and 24 hours after the start of ECMO.

Patient characteristics	BiPAP group		Control group		P value
**Overall N=9819**	**N**	**Median (IQR) or n (%)**	**N**	**Median (IQR) or n (%)**	
**Other pre-ECMO treatments**					
Narcotics	3882	3087 (79.5)	5937	4222 (71.1)	<0.001
Norepinephrine	3882	1951 (50.3)	5937	3087 (52.0)	0.092
Epinephrine	3882	141 (3.6)	5937	237 (4.0)	0.365
Vasopressin	3882	385 (9.9)	5937	562 (9.5)	0.459
Dopamine	3882	24 (0.6)	5937	32 (0.5)	0.610
Dobutamine	3882	27 (0.7)	5937	76 (1.3)	0.005
Bicarbonates	3882	291 (7.5)	5937	350 (5.9)	0.002
High frequency oscillatory ventilation	3882	54 (1.4)	5937	123 (2.1)	0.013
Inhaled anesthetics	3882	46 (1.2)	5937	67 (1.1)	0.798
Nitroprusside	3882	2 (0.1)	5937	7 (0.1)	0.288
**Co-infections at the ECMO start**					
Bacterial pneumonia	3882	1652 (42.6)	5937	2166 (36.5)	<0.001
Co-Viral infection	3882	299 (7.7)	5937	378 (6.4)	0.011
Bloodstream infection	3882	713 (18.4)	5937	867 (14.6)	<0.001
Urinary tract infection	3882	284 (7.3)	5937	420 (7.1)	0.650
**Parameters before the ECMO start**					
Peak inspiratory pressure, cmH_2O	2670	34 (30-38)	3896	33 (30-38)	<0.001
**Hemodynamics**					
Systolic blood pressure, mmHg	3335	118 (104-134)	4878	118 (104-133)	0.988
Dyastolic blood pressure, mmHg	3333	63 (56-72)	4871	63 (56-72)	0.757
Mean blood pressure, mmHg	2968	80 (71-90)	4160	80 (71-90)	0.730
Central venous O_2 saturation, %	173	70 (60-81)	246	73 (64-80)	0.171
**Parameters 24h after the ECMO start**					
**Blood gas and acid base**					
pH	3684	7.40 (7.36-7.44)	5699	7.40 (7.36-7.44)	0.591
pCO_2, mmHg	3625	46 (41-52)	5551	46 (41-52)	0.048
pO_2, mmHg	3628	72 (61-88)	5552	74 (63-92)	<0.001
FiO_2, %	3434	50 (40-90)	5312	50 (40-75)	0.014
**Ventilatory and ECMO setting**					
Respiratory rate, breaths/min	3498	12 (10-16)	5219	12 (10-15)	0.325
Positive end-expiratory pressure, cmH_2O	3477	10 (10-12)	5236	10 (10-12)	0.001
Peak inspiratory pressure, cmH_2O	3011	25 (21-29)	4504	24 (20-28)	<0.001
ECMO blood flow, L/min	3647	4.3 (3.9-4.9)	5449	4.3 (3.8-4.9)	0.004
**Hemodynamics**					
Systolic arterial pressure, mmHg	3606	118 (107-131)	5512	117 (106-131)	0.315
Dyastolic arterial pressure, mmHg	3606	60 (54-67)	5509	60 (54-68)	0.051
Mean arterial pressure, mmHg	3357	77 (70-85)	4871	77 (71-86)	0.048
Central venous O_2 saturation, %	884	71 (65-78)	1038	72 (66-79)	0.105

Data are presented as frequency and percentage for qualitative variables and as median (25th-75th percentile) for quantitative variables. BiPAP, bilevel positive airway pressure; ECMO, extracorporeal membrane oxygenation; RS: respiratory support; pCO_2, arterial carbon dioxide tension; pO_2, arterial oxygen tension; FiO_2, inspiratory oxygen fraction.

Table E3. Complications during ECMO support and patient outcomes stratified by study group.

Patient characteristics	BIPAP group		Control group		P value
**Overall N=9819**	**N**	**Median (IQR) or n (%)**	**N**	**Median (IQR) or n (%)**	
**Complications during ECMO**					
Renal replacement therapy	3882	1059 (27.3)	5937	1428 (24.1)	<0.001
Creatinine >3 mg/dl	3882	219 (5.6)	5937	256 (4.3)	0.003
Pneumothorax	3882	639 (16.5)	5937	739 (12.5)	<0.001
Circuit change	3882	504 (13.0)	5937	627 (10.6)	<0.001
Brain death	3882	41 (1.1)	5937	78 (1.3)	0.254
Seizures	3882	20 (0.5)	5937	42 (0.7)	0.240
CNS hemorrhage	3882	280 (7.2)	5937	387 (6.5)	0.181
CNS stroke	3882	77 (2.0)	5937	106 (1.8)	0.478
Gastrointestinal hemorrhage	3882	310 (8.0)	5937	401 (6.8)	0.021
Cannula bleeding	3882	231 (6.0)	5937	314 (5.3)	0.161
Pulmonary hemorrhage	3882	161 (4.2)	5937	216 (3.6)	0.199
Surgical site bleeding	3882	241 (6.2)	5937	305 (5.1)	0.024
**ECMO runs,** number	3882		5937		0.480
1		3850 (99.2)		5876 (99.0)	
2		31 (0.8)		60 (1.0)	
3		1 (0.0)		1 (0.0)	
**Total ECMO duration,** days					
Overall	3882	20 (10-35)	5937	18 (9-32)	<0.001
Discharged alive	1743	20 (10-37)	3069	16 (9-32)	<0.001
Dead	2017	21 (12-34)	2745	19 (10-32)	0.001
**Mortality during ECMO**	3860	1828 (47.4)	5887	2411 (41.0)	<0.001
**Lung transplant**	3860	57 (1.7)	5887	58 (1.2)	0.028
**Hospital length of stay,** days					
Overall	3882	35 (21-55)	5929	34 (20-54)	0.020
Discharged alive	1743	46 (30-67)	3063	42 (26-63)	<0.001
Dead	2017	27 (17-42)	2743	26 (16-41)	0.029
**Outcome at hospital discharge**	3882		5937		<0.001
Discharged alive		1743 (44.9)		3069 (51.7)	

Discharged on ECMO		122 (3.1)		123 (2.1)	
Dead		2017 (52.0)		2745 (46.2)	

Continuous data are presented as median and interquartile range, qualitative variables are presented absolute frequence (percentage). BiPAP, bilevel positive airway pressure; ECMO, extracorporeal membrane oxygenation, CNS,

Table E4. Multivariate logistic regression for hospital mortality (n. 2752 patients).

	Odds ratios (95% CI)	p-value
**Age** (year increase)	1.04 (1.03-1.05)	<0.001
**Female** (Ref. No)	0.77 (0.63-0.95)	0.015
**Race** (Ref. White)		
Asian	1.14 (0.83-1.58)	0.407
Black	1.2 (0.89-1.62)	0.229
Hispanic	1.09 (0.83-1.43)	0.527
Middle Eastern or North African	0.97 (0.62-1.53)	0.907
Multiple	1.14 (0.79-1.63)	0.487
Other	1.56 (1.02-2.39)	0.041
Unknown	1.18 (0.65-2.15)	0.584
**Admission year 2021** (Ref. 2020)	1.2 (0.97-1.49)	0.1
**ELSO Chapter** (Ref. North America)		
Asia-Pacific	0.29 (0.14-0.59)	0.001
European	0.62 (0.47-0.81)	0.001
Latin-american	0.69 (0.42-1.15)	0.152
South and West Asia	1.45 (0.88-2.41)	0.149
**Comorbidities** (Ref. No)		
Hypertension	0.77 (0.63-0.93)	0.006
Diabetes	0.99 (0.81-1.19)	0.887
Obesity (BMI of 30 kg/m^2 or more)	1 (0.82-1.22)	0.997
Cancer	2.79 (1.3-5.99)	0.009
Immunocompromised	1.85 (1.18-2.91)	0.007
Pre-existing heart disease	2.1 (1.23-3.58)	0.006
Pre-existing renal insufficiency	0.66 (0.38-1.15)	0.141
Pre-existing lung disease	1.06 (0.64-1.78)	0.817
Asthma	0.91 (0.69-1.21)	0.532
Frailty	3.9 (1.11-13.76)	0.034
Pregnancy	0.31 (0.19-0.5)	<0.001
**Co-diagnoses at the ECMO start** (Ref. No)		
ARDS	0.75 (0.58-0.95)	0.017
Septic shock	1.46 (1.12-1.9)	0.006
Cardiogenic shock	1.76 (1.09-2.82)	0.02
Pneumothorax	1.58 (1.23-2.03)	<0.001
Pneumonia	0.97 (0.8-1.17)	0.74
Myocarditis	0.7 (0.24-2)	0.502
Acute renal failure	1.67 (1.31-2.12)	<0.001
Any co-infection	0.98 (0.81-1.18)	0.804
**Treatments before ECMO** (Ref. No)		
Prone positioning	1.14 (0.93-1.39)	0.202
Neuromuscolar blocking agents	0.96 (0.76-1.21)	0.723
Inhaled nitric oxyde	0.99 (0.74-1.31)	0.924
Steroids	1.61 (1.32-1.97)	<0.001
Renal replacement therapy	0.96 (0.62-1.5)	0.868
Vasoactives	1.18 (1-1.39)	0.045

**Physiologic and clinical parameters before ECMO**		
pH (0.1 increase)	0.91 (0.83-1)	0.057
pCO_2 (10 mmHg increase)	1.09 (1.03-1.15)	0.002
pO_2/FiO_2 (10 mmHg increase)	0.98 (0.97-0.99)	0.002
Respiratory rate (breath/minute increase)	0.99 (0.98-1)	0.141
Positive end-expiratory pressure (cmH_20 increase)	1.01 (0.98-1.03)	0.522
**Referral** (Ref. not trasported**)**		
Transported not on ECMO	1.19 (0.95-1.49)	0.133
Transported on ECMO	1.43 (1.04-1.98)	0.03
**Time from hospital admission to intubation** (day increase)	1.03 (1-1.06)	0.035
**Duration of mechanical ventilation before ECMO** (day increase)	1.04 (1.01-1.06)	0.011
**Use of BiPAP** (Ref. No)	1.32 (1.08-1.61)	0.006

Risk of hospital mortality was expressed using OR with 95% CI adjusted by robust clustering taking into account the study centers of the ELSO registry (i.e. clusters). BMI, body mass index; ECMO, extracorporeal membrane oxygenation; BiPAP, bilevel positive airway pressure; ARDS, acute respiratory distress syndrome; pCO2, arterial carbon dioxide tension; pO2/FiO2, ratio of arterial oxygen tension to inspiratory oxygen fraction; BiPAP, bilevel positive airway pressure.

Table E5. Multivariate logistic regression for hospital mortality (n. 5945 patients).

	**Odds ratios (95% CI)**	**p-value**
**Age** (year increase)	1.22 (1.05-1.42)	<0.001
**Female** (Ref. No)	0.85 (0.73-0.98)	0.029
**Race** (Ref. White)		
Asian	1.07 (0.83-1.37)	0.613
Black	1.09 (0.86-1.37)	0.479
Hispanic	1.06 (0.86-1.3)	0.574
Middle Eastern or North African	0.79 (0.55-1.14)	0.208
Multiple	0.97 (0.7-1.34)	0.85
Other	1.34 (0.96-1.86)	0.084
Unknown	0.98 (0.72-1.34)	0.913
**Admission year 2021** (Ref. 2020)	1.22 (1.05-1.42)	0.008
**ELSO Chapter** (Ref. North America)		
Asia-Pacific	0.36 (0.19-0.69)	0.002
European	0.57 (0.44-0.75)	<0.001
Latin-american	0.82 (0.48-1.38)	0.446
South and West Asia	1.52 (0.92-2.49)	0.099
**Comorbidities** (Ref. No)		
Hypertension	0.92 (0.81-1.06)	0.244
Diabetes	1.02 (0.89-1.17)	0.74
Obesity (BMI of 30 kg/m^2 or more)	1.01 (0.88-1.17)	0.85
Cancer	1.56 (0.93-2.63)	0.093
Immunocompromised	1.8 (1.29-2.53)	0.001
Pre-existing heart disease	1.56 (1.06-2.3)	0.023
Pre-existing renal insufficiency	0.8 (0.52-1.25)	0.33
Pre-existing lung disease	1.13 (0.82-1.55)	0.458
Asthma	1.02 (0.85-1.23)	0.817
Frailty	2.59 (1.08-6.25)	0.034
Pregnancy	0.38 (0.27-0.54)	<0.001
**Co-diagnoses at the ECMO start** (Ref. No)		
ARDS	0.86 (0.7-1.05)	0.135
Septic shock	1.6 (1.32-1.95)	<0.001
Cardiogenic shock	1.36 (1.01-1.81)	0.04
Pneumothorax	1.54 (1.32-1.8)	<0.001
Pneumonia	0.97 (0.84-1.13)	0.695
Myocarditis	0.55 (0.2-1.53)	0.25
Acute renal failure	1.54 (1.3-1.82)	<0.001
Any co-infection	1.01 (0.88-1.15)	0.936
**Treatments before ECMO** (Ref. No)		
Prone positioning	1.1 (0.97-1.24)	0.13
Neuromuscolar blocking agents	0.95 (0.79-1.13)	0.535
Inhaled nitric oxyde	0.94 (0.76-1.18)	0.612
Steroids	1.27 (1.08-1.51)	0.004
Renal replacement therapy	0.81 (0.6-1.1)	0.175

Vasoactives	1.15 (1.01-1.32)	0.036
**Physiologic and clinical parameters before ECMO**		
pH (0.1 increase)	0.94 (0.88-1.01)	0.089
pCO_2 (10 mmHg increase)	1.09 (1.05-1.13)	<0.001
pO_2/FiO_2 (10 mmHg increase)	0.97 (0.96-0.99)	<0.001
Respiratory rate (breath/minute increase)	1 (0.99-1.01)	0.834
Positive end-expiratory pressure (cmH_20 increase)	1 (0.99-1.02)	0.828
**Referral** (Ref. not trasported**)**		
Transported not on ECMO	1.05 (0.89-1.24)	0.567
Transported on ECMO	0.98 (0.78-1.24)	0.861
**Duration of mechanical ventilation before ECMO** (day increase)	1.02 (1-1.03)	0.012
**Use of BiPAP** (Ref. No)	1.22 (1.05-1.42)	0.008

Risk of hospital mortality was expressed using OR with 95% CI adjusted by robust clustering taking into account the study centers of the ELSO registry (i.e. clusters). BMI, body mass index; ECMO, extracorporeal membrane oxygenation; BiPAP, bilevel positive airway pressure; ARDS, acute respiratory distress syndrome; pCO2, arterial carbon dioxide tension; pO2/FiO2, ratio of arterial oxygen tension to inspiratory oxygen fraction.

Table E6. Multivariate logistic regression for hospital mortality for patients who underwent BiPAP, HFNC, CPAP or no respiratory support (n. 2043 patients).

	**Odds ratios (95% CI)**	**p-value**
**Age** (year increase)	1.05 (1.03-1.06)	<0.001
**Female** (Ref. No)	0.87 (0.68-1.12)	0.284
**Race** (Ref. White)		
Asian	1.13 (0.78-1.63)	0.518
Black	1.21 (0.87-1.68)	0.266
Hispanic	1.04 (0.76-1.41)	0.825
Middle Eastern or North African	1.07 (0.64-1.78)	0.8
Multiple	1.31 (0.84-2.04)	0.236
Other	1.81 (1.07-3.07)	0.027
Unknown	1.37 (0.68-2.75)	0.384
**Admission year** (Ref. 2020)		
2021	1.24 (0.97-1.6)	0.089
**ELSO Chapter** (Ref. North America)		
Asia-Pacific	0.3 (0.14-0.66)	0.002
European	0.61 (0.45-0.83)	0.002
Latin-american	0.85 (0.53-1.37)	0.5
South and West Asia	1.29 (0.76-2.17)	0.349
**Comorbidities** (Ref. No)		
Hypertension	0.69 (0.55-0.86)	0.001
Diabetes	0.94 (0.74-1.2)	0.623
Obesity (BMI of 30 kg/m^2 or more)	0.85 (0.68-1.07)	0.164
Cancer	3.78 (1.53-9.3)	0.004
Immunocompromised	1.94 (1.15-3.28)	0.013
Pre-existing heart disease	1.69 (0.96-2.96)	0.069
Pre-existing renal insufficiency	0.62 (0.32-1.2)	0.152
Pre-existing lung disease	0.88 (0.48-1.62)	0.679
Asthma	1.03 (0.74-1.45)	0.848
Frailty	4.81 (1.43-16.19)	0.011
Pregnancy	0.26 (0.15-0.47)	<0.001
**Co-diagnoses at the ECMO start** (Ref. No)		
ARDS	0.83 (0.62-1.11)	0.21
Septic shock	1.65 (1.23-2.21)	0.001
Cardiogenic shock	1.66 (0.93-2.99)	0.089
Pneumothorax	1.55 (1.11-2.15)	0.01
Pneumonia	1.01 (0.83-1.25)	0.889
Myocarditis	0.73 (0.23-2.3)	0.591
Acute renal failure	1.61 (1.23-2.11)	0.001
Any co-infection	0.95 (0.75-1.19)	0.635
**Treatments before ECMO** (Ref. No)		
Prone positioning	1.18 (0.95-1.47)	0.141
Neuromuscolar blocking agents	0.94 (0.71-1.25)	0.684
Inhaled nitric oxyde	0.93 (0.7-1.23)	0.598

Steroids	1.62 (1.29-2.04)	<0.001
Renal replacement therapy	0.81 (0.5-1.3)	0.386
Vasoactives	1.18 (0.97-1.44)	0.106
**Physiologic and clinical parameters before ECMO**		
pH (0.1 increase)	0.87 (0.78-0.97)	0.015
pCO_2 (10 mmHg increase)	1.09 (1.02-1.16)	0.007
pO_2/FiO_2 (10 mmHg increase)	0.98 (0.96-0.99)	0.004
Respiratory rate (breath/minute increase)	0.99 (0.97-1)	0.102
Positive end-expiratory pressure (cmH$_2$0 increase)	1.01 (0.98-1.04)	0.421
**Referral** (Ref. not trasported**)**		
Transported not on ECMO	1.16 (0.88-1.52)	0.285
Transported on ECMO	1.45 (1.02-2.06)	0.037
**Time from hospital admission to intubation** (day increase)	1.04 (1-1.07)	0.036
**Duration of mechanical ventilation before ECMO** (day increase)	1.03 (1-1.06)	0.047
**Type of noninvasive respiratory support before ETI** (Ref. BiPAP)		
No respiratory support	0.59 (0.42-0.84)	0.004
Only HFNC	0.66 (0.49-0.9)	0.008
Only CPAP\	0.83 (0.56-1.22)	0.343

Risk of hospital mortality was expressed using OR with 95% CI adjusted by robust clustering taking into account the study centers of the ELSO registry (i.e. clusters). BMI, body mass index; ECMO, extracorporeal membrane oxygenation; ARDS, acute respiratory distress syndrome; pCO2, arterial carbon dioxide tension; pO2/FiO2, ratio of arterial oxygen tension to inspiratory oxygen fraction; ETI, endotracheal intubation; BiPAP, bilevel positive airway pressure; HFNC, high flow nasal cannulae; CPAP, continuous positive airway pressure.
